# Effects of warming on plant uptake of post‐fire nitrogen in an arctic heath tundra

**DOI:** 10.1111/nph.71047

**Published:** 2026-03-02

**Authors:** Wenyi Xu, Per Lennart Ambus

**Affiliations:** ^1^ College of Ecology and Environment, Co‐Innovation Center for Sustainable Forestry in Southern China Nanjing Forestry University Nanjing 210037 China; ^2^ Department of Soil and Environment Swedish University of Agricultural Sciences Uppsala 75007 Sweden; ^3^ Department of Geosciences and Natural Resource Management University of Copenhagen Copenhagen 1350 Denmark

**Keywords:** Arctic, inorganic nitrogen, nitrogen‐15 tracing, pyrogenic organic matter, shrub species, tundra fire, warming

## Abstract

- Postfire nitrogen (N) becomes increasingly important with the rising frequency of fires in arctic tundra, and climate warming is expected to accelerate plant recovery following fire. However, how plants differ in utilizing this postfire N and how their postfire N uptake responds to warming remains unknown.
- We conducted a fire experiment in combination with a warming treatment using open top chambers (OTCs) in an arctic heath tundra, West Greenland. We investigated the longer‐term fate of two postfire N forms by tracing inorganic N (^15^NH_4_^+^‐N and ^15^NO_3_^−^‐N) and pyrogenic N pools (PyOM‐^15^N) and examined how postfire N was acquired by vegetation at functional group‐ and species‐specific levels.
- Most postfire inorganic and pyrogenic ^15^N (> 67%) was lost over the 4 yr following the fire, indicating limited N fertilization effects on plant recovery. Warming increased moss aboveground biomass and thus enhanced moss uptake of PyOM‐^15^N. By contrast, warming increased the capacity of graminoids to take up inorganic ^15^N (+200%), despite their unchanged aboveground biomass.
- Our results show that warming alters postfire N cycling by shifting the pathways through which different plant functional groups access fire‐derived N, with important implications for vegetation recovery and nutrient feedbacks in a warmer, more fire‐prone Arctic.

## Introduction

Over the past half century, air temperatures in the Arctic have increased by over 3°C, more than three times the global average (Rantanen *et al*., 2022). Consequently, the frequency and extent of tundra fires has been on the rise due to the occurrence of drier and warmer summers (Lewis *et al*., 2019; McCarty *et al*., 2020). For example, an unusually severe Anaktuvuk river fire burned 1039 km^2^ of tundra on Alaska's North Slope in 2007 (He *et al*., 2021). Hitherto, wildfires rarely occurred in Greenland, but in 2017, a major fire broke out in West Greenland burning for 2 wk and scorching 1200 ha of tundra (Evangeliou *et al*., 2019). Fires are often landscape‐scale disturbances that significantly impact carbon (C) and nitrogen (N) stocks and cycling (Bowman *et al*., 2009; Wilkinson *et al*., 2023) and corresponding net greenhouse gas budgets (Hermesdorf *et al*., 2022). This is a consequence of detrimental alterations in physical (e.g. soil temperatures and moisture), chemical (e.g. nutrient availability) and biological (e.g. vegetation removal and microbial mortality) environmental conditions (Mack *et al*., 2011; Barrett *et al*., 2012; Pellegrini *et al*., 2021).

Depending on fire conditions (intensity, duration, temperature, and oxygen availability), vegetation biomass and soil organic matter (SOM) transform into ashes and pyrogenic organic matter (chars; PyOM). Nitrogen in ashes is primarily in the form of inorganic N (ammonium, NH_4_^+^‐N), whereas PyOM consists of variable fractions of pyrogenic organic N. These represent two main postfire N forms, both of which can act as an N source for both plants and microbes (Xu *et al*., 2022b). Postfire inorganic N can be assimilated by microorganisms and plants and incorporated into the soil organic N reserve, but also lost as gases, and by leaching, erosion, and runoff (Martí‐Roura *et al*., 2013; Hanan *et al*., 2016). Although N losses rather than N retention might be favored during the early stages of postfire vegetation recovery due to complete absence or low biomass of vegetation, microbes could obtain a competitive advantage and play an important role in immobilizing N (Bell & Binkley, 1989; Hollingsworth *et al*., 2013). For example, in burned prairies, the increased immobilization of N within SOM due to higher microbial N demands enhanced soil retention of potentially mineralized N, and this relatively recalcitrant N can then be recycled and become available to the recovering plants (Dell *et al*., 2005). This is a key mechanism explaining how greater productivity is maintained in N‐limited annually burned prairies (Dell *et al*., 2005). Furthermore, long‐term (12 yr) retention of postfire inorganic N in top soils has been reported in Mediterranean shrubland and grassland ecosystems, due to tight internal N recycling between plants, litter, and soil (Martí‐Roura *et al*., 2013).

The chemical composition of PyOM comprises both a recalcitrant and labile fraction. The recalcitrant fraction is primarily composed of condensed aromatic structures such as polycyclic aromatic hydrocarbons (PAHs), which are largely resistant to microbial decomposition because of their fused ring structures and low O : C ratios (Knicker, 2007). The relatively labile fraction is mainly in the form of dissolved organic compounds (Hockaday *et al*., 2006; Bostick *et al*., 2018). The initial labile fraction of PyOM may trigger an increase in postfire soil inorganic N availability by two mechanisms. Either via mineralization of N‐containing PyOM compounds or via a priming effect on native soil organic N mineralization, thus contributing to a short‐term fertilization effect on recovering plants and microbes (Caon *et al*., 2014; Soong *et al*., 2014; Maestrini *et al*., 2015). The remaining recalcitrant fraction of PyOM, on the other hand, may be partially transformed into labile forms by soil microorganisms over time, and function as a slower N‐release source, potentially impacting long‐term soil nutrient availability and primary production (Bird *et al*., 2015; Michelotti & Miesel, 2015; Soong & Cotrufo, 2015). However, a quantitative assessment of N retention and loss, plant N uptake, and microbial N assimilation in arctic tundra over a long‐term basis following fire is lacking. This is particularly important because postfire vegetation recovery in arctic tundra is usually slow, constrained by low temperatures and short growing seasons. The extent to which plants are able to acquire postfire N will largely depend on the capacity of the system (soil and microbes) to retain inorganic N and to utilize N transformed from recalcitrant PyOM‐N over time.

Plant uptake of postfire N may vary among plant functional groups and species. Deep‐rooting graminoids could be more capable of acquiring inorganic N compared with shallow rooting dwarf shrubs, since an increasing amount of relatively mobile N compounds (e.g. NO_3_^−^) has been downward leached to deeper soil layers over time after the fire (Oulehle *et al*., 2016; Wang *et al*., 2016, 2018). By contrast, dwarf shrubs could exhibit a stronger capacity to utilize N derived from pyrogenic N compared with the other functional groups, due to their association with mycorrhizal fungi that are able to decompose recalcitrant organic matter complexes (Kohler *et al*., 2015; Lindahl & Tunlid, 2015; Ward *et al*., 2022).

Temperature is a key factor for regulating plant productivity and soil biogeochemical processes in arctic ecosystems (Karhu *et al*., 2014; Xu *et al*., 2024b). Climate warming has been reported to promote vegetation recovery after fire (Hu *et al*., 2015), which may enable plants of particular functional groups or species to regain competitive advantage for N acquisition. For example, compared to graminoids and evergreen shrubs, deciduous shrubs (such as *Betula nana*) can respond more rapidly to warming due to their larger canopy leaf areas and taller plant heights (DeMarco *et al*., 2014). These traits enable them to intercept light and accumulate biomass quickly, potentially increasing their N demands and uptake through the rapid regrowth of aboveground biomass. By contrast, graminoids may increase their N uptake capacity through their thinner or deeper root systems in response to warming (Wang *et al*., 2017). In addition, many moss species are capable of recolonization and rapid growth under warmed conditions (Turetsky *et al*., 2012; Zuijlen *et al*., 2022). They effectively retain the assimilated N within their tissues and allocate it to new growth, rather than being released to the soil (Turetsky *et al*., 2012; Barthelemy *et al*., 2018). This tight internal N cycling in mosses can reduce the postfire N availability for vascular plant uptake in subsequent years. Microbial degradation and utilization of the recalcitrant PyOM‐N may also increase under warmer conditions (Xu *et al*., 2022b; Graham *et al*., 2023), thus providing a rising supply of available N such as low‐molecular‐weight organic N for plant uptake. However, it remains largely unknown how functional group‐ or species‐specific plants and microbes acquire and retain N following a fire under future warmer conditions.

In this study, we applied ^15^N‐labeled tracers (^15^N‐labeled ammonium and nitrate as well as PyOM) on the ground surface immediately after an experimental fire in an arctic dry heath tundra, West Greenland. Our aim was (1) to quantify the longer‐term fate and partitioning of inorganic N and N derived from PyOM representing two main postfire N sources and (2) to investigate how arctic tundra plants, differentiated by functional groups and species, acquire postfire N. The N losses during the fire were not considered. In addition, the postfire biogeochemical N processes were also examined in parallel experiments under simulated future warmer conditions. We hypothesize that (1) soil can exhibit long‐lasting retention of postfire inorganic N due to tight internal N cycling within the ecosystem; (2) over time microbes and plants can utilize and retain more N transformed from recalcitrant PyOM‐N; and (3) warming increases plant uptake of postfire N due to accelerated plant regrowth and enhanced plant N uptake capacity, with these effects specific to functional groups.

## Materials and Methods

### Site description

The study site is located at Blæsedalen Valley (69°16′N, 53°27′W), close to the southern shore of Disko Island, West Greenland. This valley is in the transition zone between the Low and High Arctic and has a typical Low Arctic climate (Borggaard & Elberling, 2007). Based on meteorological data (1991–2017) from the nearby Arctic Station, the annual mean air temperature is −3°C, with monthly mean temperature ranging from 8°C in July to −14°C in March. The annual mean precipitation was 418 mm in the period of 1991–2017, of which 34% was snowfall. The site lies within a discontinuous permafrost zone and the active layer is estimated to be 1.5 m deep (Blok *et al*., 2016). The soils are poorly developed and consist of basaltic rock fragments covered by a *c*. 5 cm organic surface layer. The mean annual soil temperature at 5 cm depth is −1.9°C and frozen soil conditions prevail from October to late May (D'Imperi*o et al*., 2018). The dry heath tundra in this site is dominated by low shrubs (height < 10 cm) with a composition of deciduous species including dwarf birch (*Betula nana* L.), cowberry (*Vaccinium uliginosum* L.), and gray willow (*Salix glauca* L.), and the evergreen Arctic bell‐heather (*Cassiope tetragona* (L.) D.Don). A mixture of lichens (*Cetraria islandica* (L.) Ach. and *Stereocaulon paschale* spp.) and mosses (*Tomentypnum nitens* (Hedw.) Loeske and *Aulacomnium turgidum* (Wahlenb.) Schwägr.) covers the ground.

### Preparation of pyrogenic organic matter

In summer 2016, preparation of ^15^N‐labeled PyOM began by amending two adjacent 3 × 5 m plots near the fire experimental area with a total of 0.2 g N m^−2^ containing 0.196 g ^15^N excess m^−2^. The N was applied in two equal doses of 1.0 g N as ammonium sulfate ((^15^NH_4_)_2_SO_4_; 98 atom% ^15^N) and 0.5 g N as potassium nitrate (K^15^NO_3_; 98 atom% ^15^N) dissolved in 10 l of demineralized water by using a back‐pack sprayer. The first dose was given at the beginning of growing season (July 11) and again 2 wk later (July 25). After collecting aboveground biomass (leaves, branches, and woody stems from all plant functional groups) and litter (1 month after the last labeling), the materials were air‐dried, shredded, and split into three portions for thermal conversion: torrefaction (250°C, anoxic atmosphere), pyrolysis (550°C, anoxic atmosphere), and combustion (550°C, ambient atmosphere).

### Experimental setup and design

**Fig. 1:**
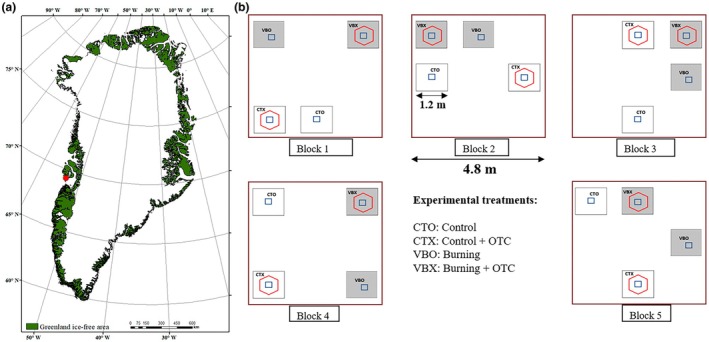
A tundra fire experiment in West Greenland. A map of Greenland (a). The location of the study site is indicated by a red dot. Experimental design overview of the tundra fire experiment in Blæsedalen, Disko Island, West Greenland (b). PyOM, pyrogenic organic matter.

In summer 2017, we conducted a tundra fire experiment within an area of 20 × 20 m on a gentle north‐east facing slope (5.7°). Two different main treatments (plot size 1.2 × 1.2 m) were included in the experiment. In one treatment, the aboveground vegetation was burned away at the site, followed by addition of a ^15^N‐inorganic N tracer (VBO). In the second treatment the aboveground biomass was cut down and removed together with surface litter, and the soil surface was scorched to simulate fire‐induced heating and then the ^15^N‐labeled PyOM was added (VRA) (Xu *et al*., 2022a,b). The two treatments were paired with experimental warming by mounting year‐round hexagonal open top chambers (OTC) upon the burning (VBX and VRX) giving a total of four treatments distributed randomly in each of five replicate blocks (block size 4.8 × 4.8 m) (Fig. 1). The OTCs were made of transparent polycarbonate, 35 cm tall and had a base diameter of 150 cm and top diameter of 85 cm (Marion, 1996), which generally increases soil temperatures by 1.0–1.5°C at 5 cm depth during summer (from June to August in 2018 (*n* = 7), 2019 (*n* = 4), and 2020 (*n* = 3); Supporting Information Table S1). On July 29, 2017, the experimental burning (VBO/VBX) was implemented using a butane‐gas weed burner at 20 cm height deployed for 7 min per plot. The 7 min were chosen after a test burning in an adjacent area for estimating the approximate duration of a natural wind‐driven fire in the area. Wildfires in arctic heath tundra are typically of low intensity due to fuel limitations, such as thin organic layers and low shrubs. Therefore, our experimental fire, conducted under prevailing weather conditions, closely resembled natural wildfires in the area. The simulation of tundra fire (simulated fire; VRA/VRX) was deployed by removing the aboveground vegetation and litter, and then scorching/heating each plot for 7 min with the butane‐gas burner, and finally adding the ^15^N‐labeled PyOM. The removal of aboveground vegetation and litter was conducted to avoid excess PyOM deposition, considering the addition of extra ^15^N‐labeled PyOM afterward. The burner was moved around within each plot area to make burning as homogenous as possible. During the burning, temperatures in the surface vegetation layer stayed above 100°C for 3–4 min in total. Soil temperatures in the top 0–2 cm increased up to 55°C and were above 30°C for 5–20 min, whereas they did not exceed 15°C at 5 cm soil depth. After the fire, aboveground vegetation was completely burned leaving chars and ashes on the soil surface, the cryptogamic cover, and litter was likewise mostly burned away, though unburned, but scorched, patches remained at the soil surface. There were no significant changes in soil C and N pools or soil pH after the fire (Xu *et al*., 2022a).

### Nitrogen‐15 labeling

To study the fate of postfire N, two ^15^N forms (inorganic ^15^N and ^15^N‐PyOM) were applied to the tundra surface after the fire. On July 30, 2017 (1 d after burning), the PyOM‐^15^N was applied to each simulated burned plot (VRA/VRX). The final amount of the applied PyOM was 372 g m^−2^ with 216 g from torrefaction (58%), 118 g from pyrolysis (31%), and 38 g from combustion (11%) for each plot. This is equivalent to 5.8 g N m^−2^ containing a total of 0.0564 g ^15^N excess m^−2^, with 62.8%, 36.8%, and 0.3% of ^15^N from torrefaction, pyrolysis, and combustion, respectively. The inorganic ^15^N was applied as a fine mist atop the plots using the backpack sprayer. The burned plots (VBO/VBX) received equal amendments of (^15^NH_4_)_2_SO_4_‐N and K^15^NO_3_‐N, both enriched at 13.5 atom% ^15^N. The total application amounted to a nonfertilizing quantity of 0.2 g N m^−2^ (containing 0.027 g ^15^N excess m^−2^) dissolved in 1 l per plot.

### Soil and plant analysis

To investigate the fate and partitioning of postfire inorganic and pyrogenic N, all ecosystem components were sampled after 4 yr (August 19, 2021). We compartmentalized the ecosystem into 24 N pools from four treatment plots (96 N pools in total), namely species‐specific leaf and stem/shoot pools (vascular plant), moss (nonvascular plant), litter, and two depth‐specific fine root, coarse root, soil, microbial biomass, total dissolved N pools, and reported them in units of grams of N m^−2^. Carbon pools of all ecosystem components (96 C pools in total) were also reported in units of grams of C m^−2^.

All aboveground plant biomass (and litter separately) was collected from a 20 × 20 cm area that was representative for the vegetation at each plot and then was sorted by species and separated into leaves and stems/shoots. Two or three replicate soil samples were taken from each plot in the 0–5.5 cm top soil (beneath litter layer and cryptogams if present), with a 4.5‐cm‐diameter auger, then were split into 0–3.5 cm and 3.5–5.5 cm soil depths. The replicate samples were subsequently mixed thoroughly into one composite sample. Roots were separated from soil samples and split into coarse (diameter > 1 mm) and fine (diameter ≤ 1 mm) roots.

Soil moisture was calculated using oven weight loss (70°C for 48 h). Subsequently, dried soil samples were finely ground by ball milling. Soil extractions were made with a moist soil to water ratio of 1 : 5 (10 g soil; 50 ml water). The soil‐water suspensions were shaken for 1 h at room temperature, then filtrated (Whatman GF/D) and kept at −18°C until analysis. Microbial biomass C and N contents as well as ^15^N concentration were determined using the chloroform fumigation‐extraction method (Brookes *et al*., 1985). To quantify soil microbial C and N, soil was fumigated by vacuum incubation with chloroform (CHCl_3_) for 24 h before extraction as described. Twenty‐five milliliters of filtered extracts of both fumigated and nonfumigated soil were subsequently freeze‐dried and then encapsulated for ^15^N‐concentration analysis. The remaining extracts were kept frozen until analysis for total dissolved N and dissolved organic C by using a TOC‐TN analyzer (Shimadzu, Kyoto, Japan). Microbial biomass C, N, and isotopic ^15^N enrichment were calculated as the difference between fumigated and nonfumigated data. A correction factor of 0.4 for extraction efficiency was applied to estimate soil microbial C, while 0.45 was used for microbial N (Christiansen *et al*., 2012; Pedersen *et al*., 2020). All plant and litter samples were dried at 70°C for 48 h and finely ground. The total C and N contents as well as ^15^N concentration from dried soil, plant materials and precipitates were determined by elemental analysis (CE1110; Thermo Electron, Milan, Italy) coupled in continuous flow mode to a Finnigan MAT Delta PLUS isotope ratio mass spectrometer (IRMS; Thermo Scientific, Bremen, Germany).

### Calculations of ^15^N recovery

To calculate the proportion of ^15^N tracer recovered in soils, roots, microbial biomass, and leaves and stems/shoots, we used the following formula:

$$\%\text{recovery}=\frac{{\text{Sample}}^{15}N\;\mathrm{APE}\times \text{Total}\;N\;\text{pool}}{{\text{Added}}^{15}N\;\text{excess}}\times 100$$

where the sample ^15^N APE is the atom percentage excess in the soils, roots, microbial biomass, and leaves and stems/shoots after subtracting the ^15^N natural abundance. Total N pool is the soil, litter, root, leaf, and stem N pool (g N m^−2^) based on soil bulk density or litter, root, leaf, and stem/shoot biomass. Leaves and stems are combined per species for the aboveground biomass samples.

### Statistics

Before analysis, data sets were checked for normal distribution and homogeneity of variance by inspecting the QQ plots and by using the Shapiro–Wilk normality test or Levene's test. We tested significant effects of ^15^N form (^15^NH_4_^+^‐N/^15^NO_3_^−^‐N and PyOM‐^15^N) and warming (ambient temperature and warmed conditions) on ^15^N recovery in bulk soil, litter, root, microbial biomass, total dissolved N, and functional form‐ or species‐specific plant (separately for each plant functional form or shrub species). Significant differences in C and N pools as well as ^15^N recovery of aboveground biomass (separately for each ^15^N form) were also tested between plant functional groups or shrub species and between ambient temperature and warming conditions. Tests were carried out by using two‐way ANOVA (type III SS) in linear mixed effects models with the lme4 and car package (Bates *et al*., 2015; Fox & Weisberg, 2019). In these tests, ^15^N form, warming and/or plant functional form or shrub species were fixed factors, and the replicate block was specified as a random factor accounting for spatial variations within the site. *Post hoc* pairwise comparisons between levels of all significant factors were then conducted using the emmeans package, with Tukey's Honestly significant differences (Tukey HSD) *P*‐value adjustment (Lenth, 2020). The significant treatment effects are based on *P* ≤ 0.05. The correlations between recoveries of inorganic ^15^N and PyOM‐^15^N in aboveground vascular plant biomass and those in fine root biomass were examined by linear regression analysis. All analyses above were performed using R software v.4.1.2 (St Pierre *et al*., 2019).

## Results

### Partitioning of inorganic and pyrogenic N

**Fig. 2:**
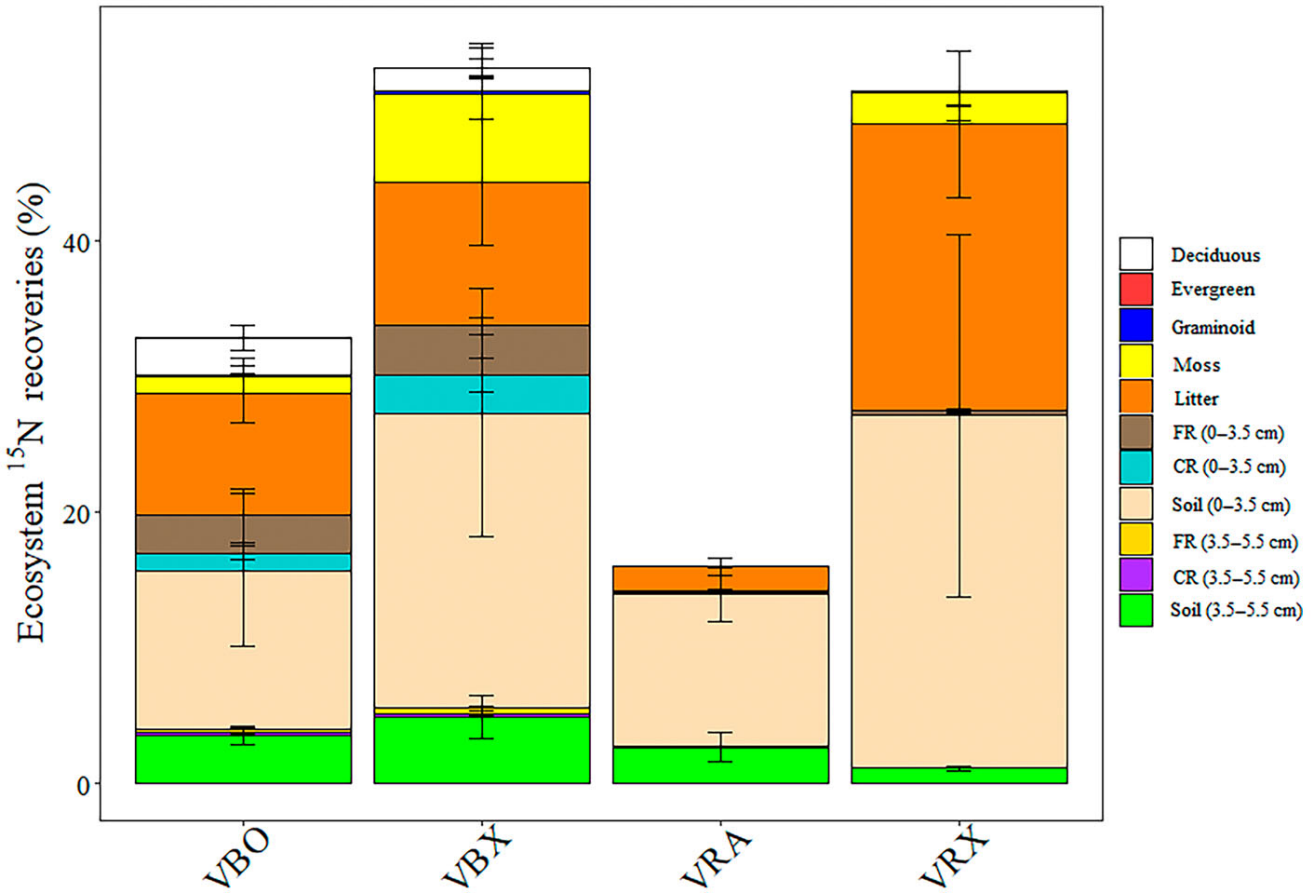
The fate of postfire nitrogen in arctic heath tundra. Partitioning of inorganic ^15^N and ^15^N‐labeled pyrogenic organic matter (PyOM‐^15^N) in deciduous shrub, evergreen shrub, graminoid, moss, litter and in coarse root (CR), fine root (FR), and bulk soil pools from 0 to 3.5 cm and from 3.5 to 5.5 cm soil depths 4 yr after the (simulated) fire under ambient temperature (VBO, VRA) and warmed conditions (VBX, VRX) in an arctic heath tundra. Error bars are SE.

All warming treatments were conducted on the burned plots. Four years after the fire, a total of 32.9 ± 9.2% and 53.8 ± 12.6% of applied ^15^NH_4_^+^‐N and ^15^NO_3_^−^‐N was recovered under ambient temperature (VBO) and warmed conditions (VBX), respectively (nonsignificant; Fig. 2; Table S2; Dataset S1). Nitrogen‐15 recovered in the bulk soil (in combined soil depths) dominated, with 14.0 ± 6.2% and 27.1 ± 10.5% of applied ^15^N under ambient (VBO) and warmed conditions (VBX), respectively (nonsignificant; Fig. 2; Table S2). Plant biomass (roots and aboveground live biomass) contained 8.6 ± 2.7% and 16.5 ± 2.7% of ^15^N, whereas only 0.46 ± 0.2% and 0.96 ± 0.6% of ^15^N was incorporated into soil microbial biomass (in combined soil depths), under ambient temperature (VBO) and warmed conditions (VBX), respectively (nonsignificant; Fig. 2; Table S2).

Total PyOM‐^15^N recovery was 16.1 ± 2.2% and 50.8 ± 12.1% under ambient (VRA) and warmed conditions (VRX), respectively, indicating a major loss of PyOM‐^15^N over 4 yr (Fig. 2; Table S2). Similar to inorganic ^15^N, the bulk soil (in combined soil depths) constituted also a major reservoir of PyOM‐^15^N, with 14.0 ± 3.0% and 27.1 ± 13.6% of applied PyOM‐^15^N under ambient temperature and warmed conditions, respectively (nonsignificant; Fig. 2; Table S2). Uptake into combined soil microbial and plant biomass (roots and aboveground live biomass) was a minor fate, only accounting for 0.38 ± 0.14% and 1.93 ± 1.31% of PyOM‐^15^N under ambient temperature and warmed conditions, respectively (*P* < 0.05; Fig. 2; Table S2).

At both soil depths, the fine and coarse roots recovered significantly more applied inorganic ^15^N than PyOM‐^15^N. Under ambient temperature conditions, fine roots had 1290% (0–3.5 cm) and 1251% (3.5–5.5 cm) higher recovery, swhereas coarse roots had 1240% (0–3.5 cm) and 7454% (3.5–5.5 cm) higher recovery (*P* < 0.05; Fig. 2; Table S2). Under warmed conditions, fine roots showed 1063% (0–3.5 cm) and 1629% (3.5–5.5 cm) higher recovery, whereas coarse roots had 3033% (0–3.5 cm) higher recovery (*P* < 0.05; Fig. 2; Table S2). There was a lower amount of PyOM‐^15^N than inorganic ^15^N incorporated into microbial biomass at 3.5–5.5 cm soil depth under warmed conditions (*P* = 0.023) and into aboveground live plant biomass under both ambient temperature (*P* < 0.01) and warmed conditions (*P* = 0.01; Fig. 2; Table S2). Although warming did not affect fine root biomass, it significantly increased fine root inorganic ^15^N uptake at 0–3.5 cm and 3.5–5.5 cm soil depths (+29% and +114%; *P* = 0.021 and *P* = 0.042, respectively; Fig. 2; Table S2). In accordance with the higher aboveground live biomass (*P* = 0.04; Fig. S1), a greater amount of PyOM‐^15^N was recovered in the aboveground biomass under warmed than under ambient temperature conditions (+3142%; *P* < 0.01; Fig. 2; Table S2).

### Warming effects on functional group‐specific uptake of inorganic and pyrogenic N

**Fig. 3:**
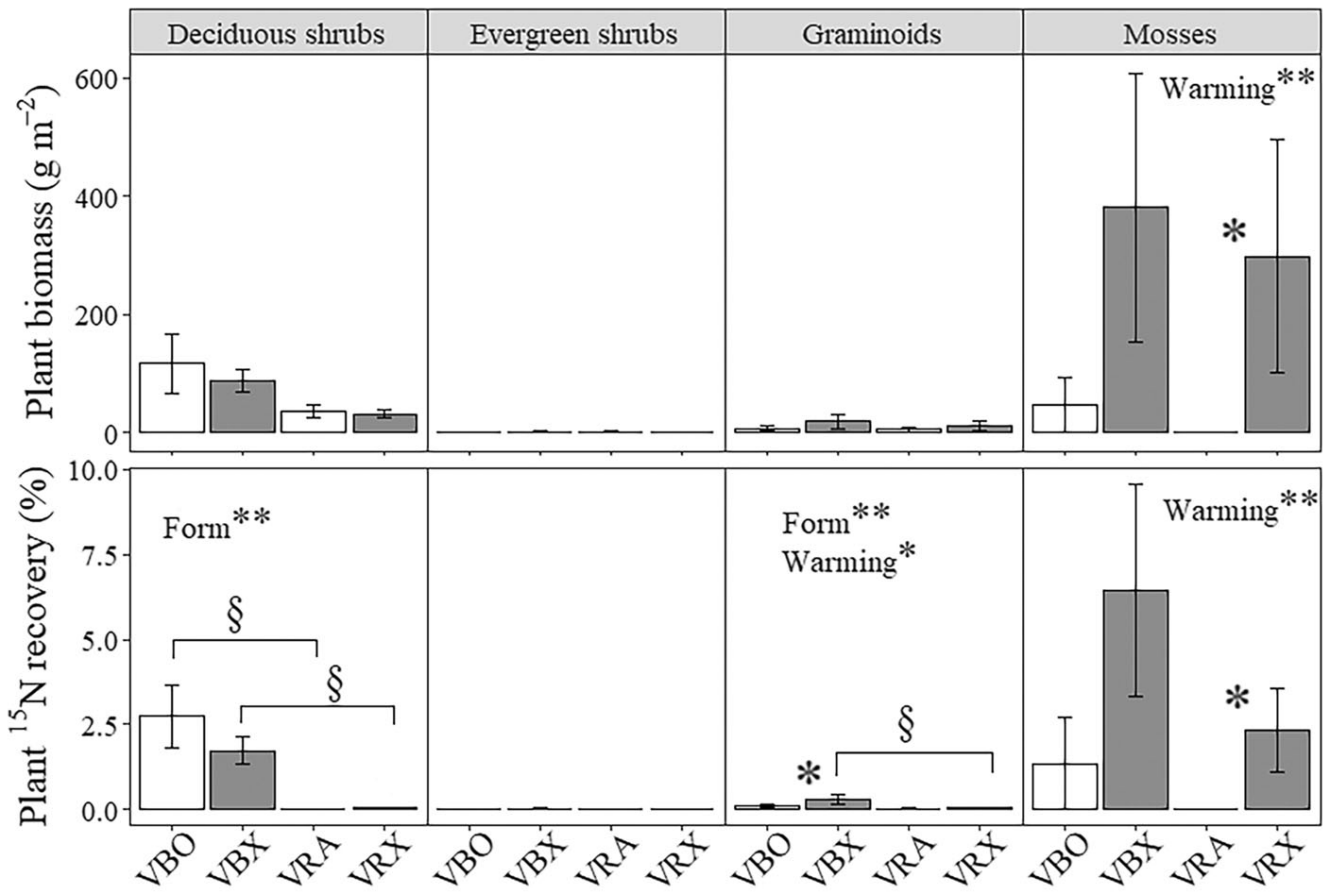
Functional group‐specific plant biomass and plant uptake of inorganic ^15^N and ^15^N‐labeled pyrogenic organic matter (PyOM‐^15^N) 4 yr after the (simulated) fire under ambient temperature (VBO, VRA) and warmed conditions (VBX, VRX). Error bars are SE. Significant differences between ambient temperature and warmed plots are shown as *, *P* ≤ 0.05, and significant differences between ^15^N forms are shown as §, *P* ≤ 0.05. Significant effects of ^15^N form and warming are shown as **, *P* ≤ 0.01.

Under ambient temperature conditions, deciduous shrubs (118 ± 50 g m^−2^) had significantly higher aboveground plant biomass (as well as biomass C and N pools) compared to the other three plant functional groups (0 ± 0, 8 ± 4 and 47 ± 47 g m^−2^ for evergreen shrubs, graminoids, and mosses, respectively; *P* < 0.05), whereas under warmed conditions, mosses (298 ± 197 g m^−2^) dominated the aboveground plant biomass (Figs 3, S2). In accordance with the regrowth patterns among plant functional groups, significantly higher uptake of inorganic ^15^N was observed in the deciduous shrubs (2.7 ± 0.9%) than the other groups under ambient temperature conditions (0 ± 0%, 0.1 ± 0% and 1.4 ± 1.4% for evergreen shrubs, graminoids, and mosses, respectively; *P* < 0.05; Fig. 3). Under warmed conditions, higher amounts of inorganic ^15^N and PyOM‐^15^N were recovered in the aboveground biomass of mosses (6.4 ± 3.1% and 2.3 ± 1.2%; *P* = 0.03 and *P* < 0.01, respectively) and deciduous shrubs (1.7 ± 0.4% and 0.1 ± 0%; *P* < 0.01) compared with evergreen shrubs (0.02 ± 0.02% and 0 ± 0%; Fig. 3).

Deciduous shrubs took up more inorganic ^15^N than PyOM‐^15^N under ambient temperature (+14 663%; *P* < 0.01), while both deciduous shrubs and graminoids took up more inorganic ^15^N than PyOM‐^15^N under warmed conditions (+3326% and +814%; for deciduous shrubs and graminoids respectively; *P* < 0.01; Fig. 3; Table S3). Warming significantly increased inorganic ^15^N uptake by graminoids (+227%; *P* = 0.023), and PyOM‐^15^N uptake by mosses (*P* = 0.039) due to their increased aboveground biomass (*P* = 0.031; Fig. 3; Table S3).

### Warming effects on shrub species‐specific uptake of inorganic and pyrogenic N

**Fig. 4:**
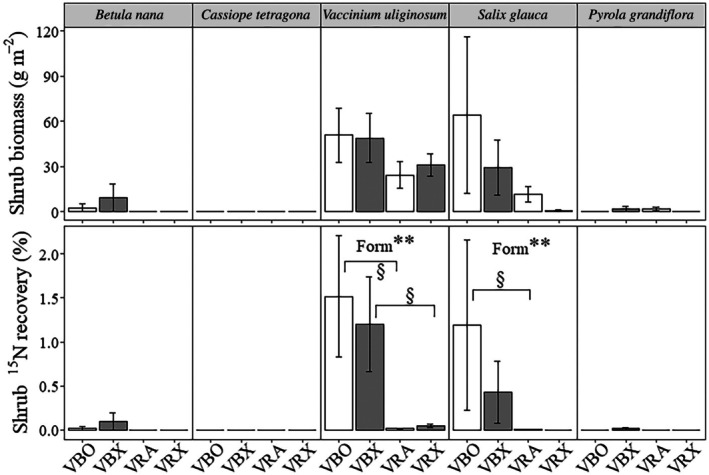
Species‐specific shrub biomass and shrub uptake of inorganic ^15^N and ^15^N‐labelled pyrogenic organic matter (PyOM‐^15^N) 4 yr after the (simulated) fire under ambient temperature (VRA, VBO) and warmed conditions (VBX, VRX). Deciduous (*Betula nana*, *Vaccinium uliginosum*, and *Salix glauca*) and evergreen (*Cassiope tetragona* and *Pyrola grandiflora*) shrubs are presented. Error bars are SE. Significant differences between ^15^N forms are shown as §, *P* ≤ 0.05. Significant effects of ^15^N form are shown as **, *P* ≤ 0.01.

Under ambient temperature conditions, the aboveground biomass (as well as biomass C and N pools) of *V. uliginosum* and *S. glauca* was significantly higher than the other three shrub species (*P* < 0.05; Figs 4, S3). Under warmed conditions, *V. uliginosum* had significantly higher aboveground biomass (*P* < 0.05), whereas *S. glauca* showed comparable biomass to the other three shrub species (Fig. 4). In accordance with the regrowth patterns among shrub species, more inorganic ^15^N was recovered in the aboveground biomass of *V. uliginosum* (1.5 ± 0.7%) and *S. glauca* (1.2 ± 1.0%) than the other shrub species under ambient temperature conditions (*P* < 0.05; Fig. 4). Under warmed conditions, *V. uliginosum* (1.2 ± 0.5%) showed higher recovery of inorganic ^15^N in its aboveground biomass than the other four shrub species (*P* < 0.05; Fig. 4). A greater amount of PyOM‐^15^N was also recovered in the aboveground biomass of *V. uliginosum* (0.01 ± 0.01% and 0.05 ± 0.02%) than the other species under both ambient temperature and warmed conditions (*P* < 0.05; Fig. 4). Under ambient conditions, *V. uliginosum* and *S. glauca* took up significantly more inorganic ^15^N than PyOM‐^15^N (+11 138% and +23 843%, respectively; *P* < 0.05; Fig. 4; Table S4). Under warmed conditions, *V. uliginosum* also showed greater uptake of inorganic ^15^N than PyOM‐^15^N (+2305%; *P* < 0.01; Fig. 4; Table S4).

### Correlations between ^15^N recovery in aboveground biomass and fine roots

**Fig. 5:**
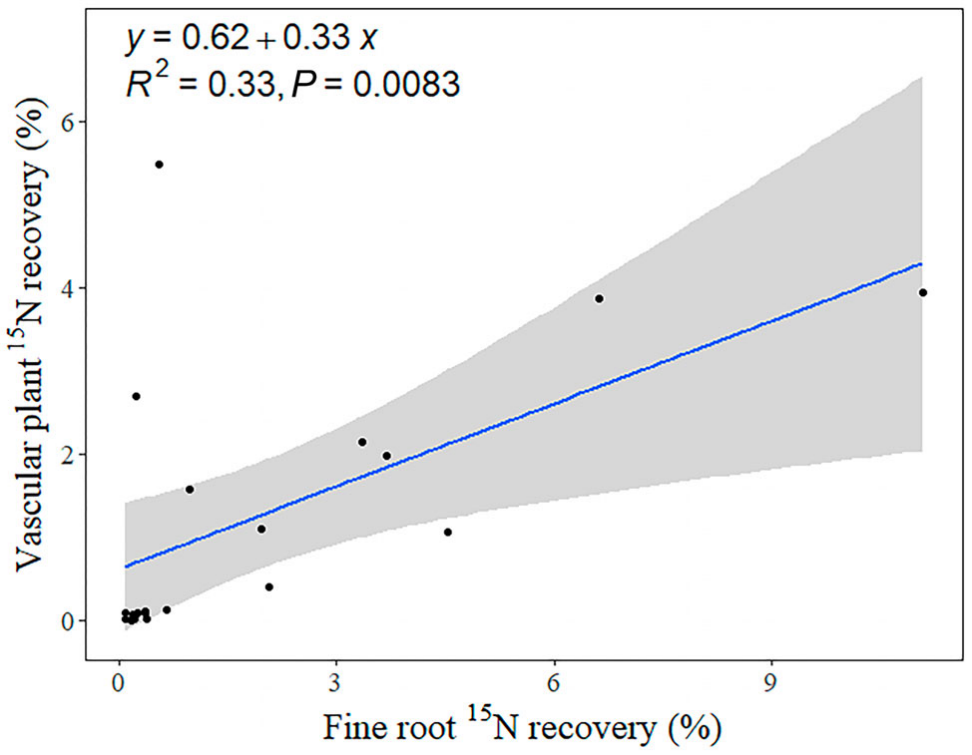
Relationship between recovery of inorganic ^15^N and ^15^N‐labeled pyrogenic organic matter (PyOM‐^15^N) in aboveground vascular plant biomass and in fine root biomass. Solid line indicates the best‐fitting linear regression line and shaded areas indicate the confidence interval (95% confidence) around the regression line.

Recovery of inorganic ^15^N and PyOM‐^15^N in aboveground vascular plant biomass (graminoids, deciduous, and evergreen shrubs) also correlated with ^15^N recovery in fine roots across treatments (in combined depths; *R* ^2^ = 0.33; *P* < 0.01; Fig. 5). Inorganic ^15^N recovery in aboveground biomass of graminoids was significantly correlated with inorganic ^15^N recovery in fine roots at 3.5–5.5 cm depth across ambient temperature and warmed conditions (*R* ^2^ = 0.76; *P* < 0.01), but not at 0–3.5 cm soil depth (Fig. S4).

## Discussion

### Longer‐term fate of postfire N

This study showed that up to 33% of inorganic N and PyOM‐N made available upon fire events was retained in the tundra ecosystem 4 yr later. This, on the other hand, means that at least 67% of these N pools was lost during the same period. In a previous study, we investigated the potential gaseous N losses from this site, and observed no ^15^N enrichment of emitted N_2_O in this dry heath tundra (Xu *et al*., 2022a,b). Hence, the low total ecosystem recovery of inorganic ^15^N and PyOM‐^15^N 4 yr after the fire was likely due to N losses caused by erosion or downward and downslope transport (Cotrufo *et al*., 2016; Santín *et al*., 2016). The lack of and slow recovery of postfire aboveground vegetation (Xu *et al*., 2024a), weakens the importance of vegetation in retaining inorganic N and increases the potential for off‐site losses of mobile N compounds (e.g. NO_3_^−^). Moreover, most PyOM has been reported to occur in the free and occluded low‐density particulate OM fractions that facilitate PyOM losses by, for example, surface run‐off (López‐Martín *et al*., 2018).

Our earlier results showed that soil inorganic ^15^N recovery remained unchanged (30%) at 0–3.5 cm depth over 2 yr after the fire, while in control plots it was decreased and significantly lower than that in burned plots. This suggested that fire increased soil N retention (Table S5). However, 4 yr after the fire, soil inorganic ^15^N recovery at 0–3.5 cm depth has decreased to 11.7%, rejecting our hypothesis (1) that soil can exhibit long‐lasting retention of postfire inorganic N. This contrasts with observations by Martí‐Roura *et al*. (2013) who found long‐term (12 yr) retention of postfire inorganic N in top soils in Mediterranean shrubland and grassland. They explained that a severe drought between 6 and 12 yr after the fire promoted plant and litter deposition and a significant amount of N derived from postfire inorganic N has been recycled (Martí‐Roura *et al*., 2013). Due to low temperatures in arctic tundra, slow decomposition of plant litter has restricted N recycling from plants to soil, causing the retention of part of the fire‐derived inorganic N in litter layer (8.9%). The inorganic ^15^N incorporated into microbial biomass at 0–3.5 cm depth has declined significantly, from 4.5% 2 yr after the fire to 0.3% 4 yr after the fire (Xu *et al*., 2022a). This suggests that microbial assimilation of fire‐derived inorganic N has decreased over time, potentially contributing to the diminished retention of inorganic N in the soil. Previous studies have demonstrated that microbial N immobilization can play a key role in postfire soil inorganic N retention in tight N‐cycling ecosystems (e.g. prairies), due to greater microbial N demands in the presence of low‐quality fire‐derived substrates (Dell *et al*., 2005; Goodridge *et al*., 2018). Alternatively, the reduced soil inorganic ^15^N recovery might be due to the increased N losses caused by intense precipitation events (Xu & Ambus, 2025). The root inorganic ^15^N recovery (fine and coarse roots) at 0–3.5 cm soil depth has increased from 2% to 4% 4 yr after the fire (Tables S5). This is likely because soil microbes were efficient competitors for N in the short term, whereas plants acquired available N over longer time scales due to their slower turnover and longer tissue longevity than soil microbes (Nordin *et al*., 2004; Clemmensen *et al*., 2008). Our earlier results of ^15^N enrichment in shrub leaves and current data both showed the contrasting postfire N uptake patterns among the shrub species (Table S6). Specifically, *V. uliginosum* can benefit more from this postfire N compared with the other shrub species. However, total live plant biomass recovered 8.6% of inorganic ^15^N 4 yr after the fire, indicating that a low amount of postfire inorganic N can be utilized and retained by plants. This is in contrast with observations by Goodridge *et al*. (2018) who reported that 52% of wildfire N could be accounted for in plant and soil microbial growth 1 yr after the fire in a chaparral watershed. These differing observations can be attributed to low microbial assimilation and short‐term soil retention of post‐fire N in the arctic tundra, which are insufficient to prevent postfire N losses and to sustain N availability during the slow plant recovery process.

Only a marginal proportion of PyOM‐^15^N was incorporated into microbial (0.02%) and plant biomass (0.35%) 4 yr after the fire. The degradability and availability of PyOM depends on its precursor material and the charring temperature (Bird *et al*., 2015; Torres‐Rojas *et al*., 2020). Since 58% of PyOM‐^15^N was produced at 250°C, PyOM‐N might be expected to be relatively less recalcitrant compared with other studies (PyOM charred at 450°C) (Singh *et al*., 2014; Fang *et al*., 2018). The ash fraction (produced from complete combustion) that contained labile inorganic N, accounted for 0.3% of applied PyOM‐^15^N. These altogether suggest that microbes and plants can utilize only a very limited amount of N transformed from recalcitrant PyOM‐N over time other than the initial labile inorganic N (ash) fraction, rejecting hypothesis (2). This is confirmed by our earlier results that microbial biomass and roots recovered 0.4% and 0.02% of ^15^N‐PyOM, respectively, 21 d after the fire (Table S5), when the loss of initial labile N fraction was assumed to be lower compared with that after 4 yr (Xu *et al*., 2022b). In addition to chemical recalcitrance, other processes may have further limited PyOM‐N availability to plants. For example, sorption of N compounds onto mineral or PyOM surfaces can reduce their mobility and accessibility to roots (Jilling *et al*., 2018; Hestrin *et al*., 2019).

### Warming effects on plant uptake of postfire N

Our results support hypothesis (3) that warming increases plant uptake of postfire N due to accelerated plant regrowth and enhanced plant N uptake capacity, with these effects specific to functional groups.

Fine root inorganic ^15^N recovery significantly increased due to warming, despite unchanged fine root biomass. This suggests that warmer temperatures enhanced capacity of roots to take up postfire N. Fine roots play a critical role in nutrient acquisition and in ensuring rapid plant regeneration by taking advantage of favorable postfire environmental conditions (Zadworny *et al*., 2016). However, root growth in arctic tundra is inhibited by low soil temperatures, potentially limiting the ability of plants to access nutrient‐rich substrates (especially N) following a fire (Bret‐Harte *et al*., 2013). A temperature increase may alter morphology of fine roots and enhance production of roots with small diameters, high specific root length and low root tissue density, leading to longer and thinner roots believed to have higher capacity for N acquisition (Poorter *et al*., 2012; Zadworny *et al*., 2016, 2017). Moreover, plants that survive a fire typically allocate sufficient resources to belowground and invest in root nutrient reserves, which are subsequently mobilized to facilitate rapid shoot regrowth (Varma *et al*., 2018; Le Stradic *et al*., 2021). This is supported by the strong relationship between aboveground vascular plant ^15^N recovery and fine root ^15^N recovery (Fig. 5). Moreover, increased PyOM‐^15^N incorporated in plant biomass (to 1.82%) due to warming (much higher than the labile inorganic fraction of PyOM), suggests a link between degradation of relatively recalcitrant N fraction of PyOM and plant utilization. The recalcitrant heterocyclic N that dominates in charred plant materials has been reported to be degradable in several incubation studies (de la Rosa & Knicker, 2011; Hilscher & Knicker, 2011; Santos *et al*., 2012; Knicker *et al*., 2013). For example, Hilscher & Knicker (2011) reported an increase in the ratio of amide to heterocyclic N ratio for grass‐derived PyOM after a 28‐month incubation period. They proposed that this change could be explained by the microbial assimilation of mineralized N originating from heterocyclic N (Hilscher & Knicker, 2011). Thus, we suggest a similar pathway in the current harsh environment, and that warming further catalyzes the degradation of PyOM‐N, making the N available to recovering plants. Considering fast turnover of microbial biomass, plants could capture extra PyOM‐N that is previously incorporated into microbial biomass and subsequently released.

Under warmed conditions, mosses dominated the retention of inorganic ^15^N, which was attributed to their high aboveground biomass and N demands. Similarly, under warmed conditions, mosses were the largest sink of PyOM‐^15^N among the plant functional groups, and their PyOM‐^15^N uptake significantly increased due to increased aboveground biomass. As plants grow larger and accumulate more biomass, their N demands increase proportionally. This is because higher biomass requires more N to support essential metabolic processes, maintain healthy tissues, and drive both reproduction and further growth (Leghari *et al*., 2016). Many moss species develop sporophytes on lateral branches and grow independently of sexual reproduction, which is a competitive growth advantage in stable environments (such as warmed and sheltered conditions) (Elmendorf *et al*., 2012; Turetsky *et al*., 2012; Zuijlen *et al*., 2022). Due to a high nutrient recycling and storage ability of mosses, most assimilated ^15^N was allocated to new growth rather than being released to the soil and being available for plant root uptake in the following years (Yano *et al*., 2010; Turetsky *et al*., 2012; Barthelemy *et al*., 2018). Thus, under future warmer conditions, mosses can benefit more from the post‐fire N than the other plant functional groups, giving them a potential advantage in adapting to post‐fire environments.

Although warming had minor effects on the regrowth of graminoids, it significantly increased the recovery of inorganic ^15^N in graminoid aboveground biomass. This suggests that warming enhanced the capacity of graminoids to take up and retain potsfire inorganic N. Previous studies have shown that graminoids are more efficient to acquire N from deeper soil than from shallower soil layers, while dwarf shrubs are more capable of utilizing N from shallower soil depths (Oulehle *et al*., 2016). Moreover, warming may accelerate nitrification and thus cause more NO_3_^−^ in soil solution (Kolstad *et al*., 2021; Xu *et al*., 2023), which potentially increases the downward leaching of N in this well‐drained tundra due to a higher mobility of NO_3_^−^ than NH_4_^+^. As a result, under warmed conditions, graminoids with their deep roots may gain a competitive advantage and become more effective in accessing and taking up postfire inorganic N from deeper soils layers (Wang *et al*., 2018). This is supported by the significant relationship between inorganic ^15^N recovery in graminoids and in fine roots at 3.5–5.5 cm soil depth (Fig. S4).

### Plant preferential uptake of postfire N forms

Both plants and soil microbes showed a preference for the uptake and utilization of postfire inorganic N rather than pyrogenic N. This preference is due to the high bioavailability, solubility, and low metabolic cost of inorganic N, which can be rapidly absorbed and used for growth and metabolism (Nicholas, 1963; Xu *et al*., 2012). By contrast, pyrogenic N is chemically recalcitrant, structurally complex, and often relatively inaccessible to most microbes, requiring specialized enzymatic breakdown and offering low nutritional benefit (Knicker, 2007; Bird *et al*., 2015; Torres‐Rojas *et al*., 2020). Graminoids and deciduous shrubs such as *V. uliginosum* and *S. glauca* primarily take up and utilize postfire inorganic N. They possess fast root uptake kinetics and often have deep and dense fine roots (graminoids) or root mycorrhizal associations (dwarf shrubs) that help them to quickly access and assimilate available N (Wang *et al*., 2018; Averill *et al*., 2019). Furthermore, the growth strategy of deciduous shrubs emphasizes rapid aboveground biomass production, which needs a large and timely supply of N to support leaf‐out and shoot growth (Sweet *et al*., 2014; Berner *et al*., 2018). Following a low‐intensity fire, *V. uliginosum* and *S. glauca* were able to rapidly re‐sprout due to the presence of surviving woody stems (Hollingsworth *et al*., 2013; Hermesdorf *et al*., 2022). This fast regrowth further increases N demands, making available postfire inorganic N the ideal source.

### Conclusion

To our best knowledge, this is the first detailed study in arctic heath tundra to quantify the long‐term fate and partitioning of postfire N across all ecosystem compartments, under ambient temperature as well as future warmer conditions. Most of the applied postfire inorganic ^15^N and pyrogenic ^15^N was lost through the 4‐yr experimental duration. Plants and microbes were able to utilize and retain only a low amount of inorganic N (9.1%) and only a marginal amount of pyrogenic N (0.39%). Warming increased plant uptake of both inorganic and pyrogenic N not only by accelerating plant regrowth but also by enhancing N acquisition capacity. Furthermore, the positive warming effects, along with the underlying mechanisms (i.e. accelerating plant regrowth and enhancing N acquisition capacity), were specific to functional groups. Our results suggest that in the longer term, fire had limited fertilization effects on arctic plant recovery. However, under a future warmer climate, postfire N will become an increasingly important N source. This shift is due not only to a rise in tundra fire events but also to enhanced N utilization by recovering plants. Overall, this study highlights the importance of investigating long‐term fate and cycling of postfire N in arctic tundra ecosystems. It also underscores the necessity of explicitly considering the combined effects of future climate warming and fire when assessing future ecosystem‐climate feedbacks. The combined effects imply that future tundra N dynamics will be governed by the balance between warming‐enhanced recovery and fire‐induced disruption, with outcomes strongly influenced by vegetation composition and plant functional traits.

## Competing interests

None declared.

## Author contributions

WX and PA performed experiments and conducted fieldwork or lab work. WX analyzed and interpreted data and wrote the manuscript. WX and PA reviewed the manuscript. PA planned and designed the research.

## Disclaimer

The New Phytologist Foundation remains neutral with regard to jurisdictional claims in maps and in any institutional affiliations.

## Supporting information


**Dataset S1** Partitioning of inorganic ^15^N and ^15^N‐labeled pyrogenic organic matter (PyOM‐^15^N).

**Fig. S1** Biomass of plants by functional forms (deciduous shrubs, evergreen shrubs, graminoids, mosses) and of coarse (CR) and fine roots (FR) from 0 to 3.5 cm and from 3.5 to 5.5 cm soil depths 4 yr after the fire under ambinent temperature (VBO) and warmed conditions (VBX).
**Fig. S2** Carbon and nitrogen contents in plants by functional forms (deciduous shrubs, evergreen shrubs, graminoids, mosses) and in bulk soil, coarse (CR) and fine roots (FR) from 0 to 3.5 cm and from 3.5 to 5.5 cm soil depths 4 yr after the fire under ambient temperature (VBO) and warmed conditions (VBX).
**Fig. S3** Species‐specific shrub biomass carbon (C) and nitrogen (N) 4 yr after the fire under ambient temperature (VBO) and warmed conditions (VBX).
**Fig. S4** Relationship between graminoid inorganic ^15^N recovery and fine root inorganic ^15^N recovery at 0–3.5 cm and 3.5–5.5 cm soil depths.
**Table S1** Seasonal averages (mean ± SE) of mannually measured soil temperature and soil moisture at 5 cm soil depth during 4 yr after the (simulated) fire under ambient termperature (VBO, VRA) and warmed conditions (VBX, VRX; 2018 *n* = 7; 2019 *n* = 4; 2021 *n* = 3).
**Table S2** Recovery of inorganic ^15^N and ^15^N‐labeled pyrogenic organic matter (PyOM‐^15^N) in various ecosystem compartments 4 yr after the (simulated) fire under ambient temperature (VBO, VRA) and warmed conditions (VBX, VRX).
**Table S3** Results of the mixed models of warming and ^15^N form effects on functional group‐specific ^15^N recovery.
**Table S4** Results of the mixed models of warming and ^15^N form effects on shrub‐specific ^15^N recovery.
**Table S5** Recovery of inorganic ^15^N and ^15^N‐labeled pyrogenic organic matter (PyOM‐^15^N) in the bulk soil and root pools from 0 to 3.5 cm soil depth during 2 yr after the (simulated) fire under ambient temperature (VBO, VRA) and warmed conditions (VBX, VRX).
**Table S6** Enrichment of inorganic ^15^N and ^15^N‐labeled pyrogenic organic matter (PyOM‐^15^N) in the leaves of the four dominant shrub species during 3 yr after the (simulated) fire under ambient temperature (VBO, VRA) and warmed conditions (VBX, VRX).
Please note: Wiley is not responsible for the content or functionality of any Supporting Information supplied by the authors. Any queries (other than missing material) should be directed to the *New Phytologist* Central Office.

## Data Availability

The data that support the findings of this study are available in the Supporting Information of this article (Dataset S1).
