# Supplementary material for: Effects of warming on plant uptake of post‐fire nitrogen in an arctic heath tundra
Source: New Phytol. 2026 Mar 2;250(3):1510–21. doi: 10.1111/nph.71047 (PMC13062696; doi:10.1111/nph.71047)
Supplement: Supplementary file 2 — Fig. S1 Biomass of plants by functional forms (deciduous shrubs, evergreen shrubs, graminoids, mosses) and of coarse (CR) and fine roots (FR) from 0 to 3.5 cm and from 3.5 to 5.5 cm soil depths 4 yr after the fire under ambinent temperature (VBO) and warmed conditions (VBX). Fig. S2 Carbon and nitrogen contents in plants by functional forms (deciduous shrubs, evergreen shrubs, graminoids, mosses) and in bulk soil, coarse (CR) and fine roots (FR) from 0 to 3.5 cm and from 3.5 to 5.5 cm soil depths 4 yr after the fire under ambient temperature (VBO) and warmed conditions (VBX). Fig. S3 Species‐specific shrub biomass carbon (C) and nitrogen (N) 4 yr after the fire under ambient temperature (VBO) and warmed conditions (VBX). Fig. S4 Relationship between graminoid inorganic 15N recovery and fine root inorganic 15N recovery at 0–3.5 cm and 3.5–5.5 cm soil depths. Table S1 Seasonal averages (mean ± SE) of mannually measured soil temperature and soil moisture at 5 cm soil depth during 4 yr after the (simulated) fire under ambient termperature (VBO, VRA) and warmed conditions (VBX, VRX; 2018 n = 7; 2019 n = 4; 2021 n = 3). Table S2 Recovery of inorganic 15N and 15N‐labeled pyrogenic organic matter (PyOM‐15N) in various ecosystem compartments 4 yr after the (simulated) fire under ambient temperature (VBO, VRA) and warmed conditions (VBX, VRX). Table S3 Results of the mixed models of warming and 15N form effects on functional group‐specific 15N recovery. Table S4 Results of the mixed models of warming and 15N form effects on shrub‐specific 15N recovery. Table S5 Recovery of inorganic 15N and 15N‐labeled pyrogenic organic matter (PyOM‐15N) in the bulk soil and root pools from 0 to 3.5 cm soil depth during 2 yr after the (simulated) fire under ambient temperature (VBO, VRA) and warmed conditions (VBX, VRX). Table S6 Enrichment of inorganic 15N and 15N‐labeled pyrogenic organic matter (PyOM‐15N) in the leaves of the four dominant shrub species during 3 yr after the (simulated) fi [file NPH-250-1510-s001.pdf]

New Phytologist Supporting Information

Article title: Effects of warming on plant uptake of post-fire nitrogen in an arctic heath tundra

Authors: Wenyi Xu, Per Lennart Ambus

Article acceptance date: 29 January 2026

The following Supporting Information is available for this article:

Table S1. Seasonal averages (mean ± standard error) of mannually measured soil temperature and soil moisture at 5 cm soil depth during four years after the (simulated) fire under ambient termperature (VBO, VRA) and warmed conditions (VBX, VRX; 2018 n = 7; 2019 n = 4; 2021 n = 3).

	VBO	VBX	VRA	VRX
Soil temperature				
2018	8.6±0.2	9.6±0.3	10.0±0.6	10.9±0.5
2019	10.5±0.9	11.4±1.0	10.8±0.9	11.9±0.5
2021	10.7±0.2	12.0±0.4		
Soil moisture				
2018	30.2±2.8	34.7±3.6	30.3±4.3	35.0±5.2
2019	18.7±1.7	19.5±2.0	19.7±2.9	18.6±2.6
2021	22.5±0.8	22.6±0.9		

No available data for 2021 from VRA and VRX plots.

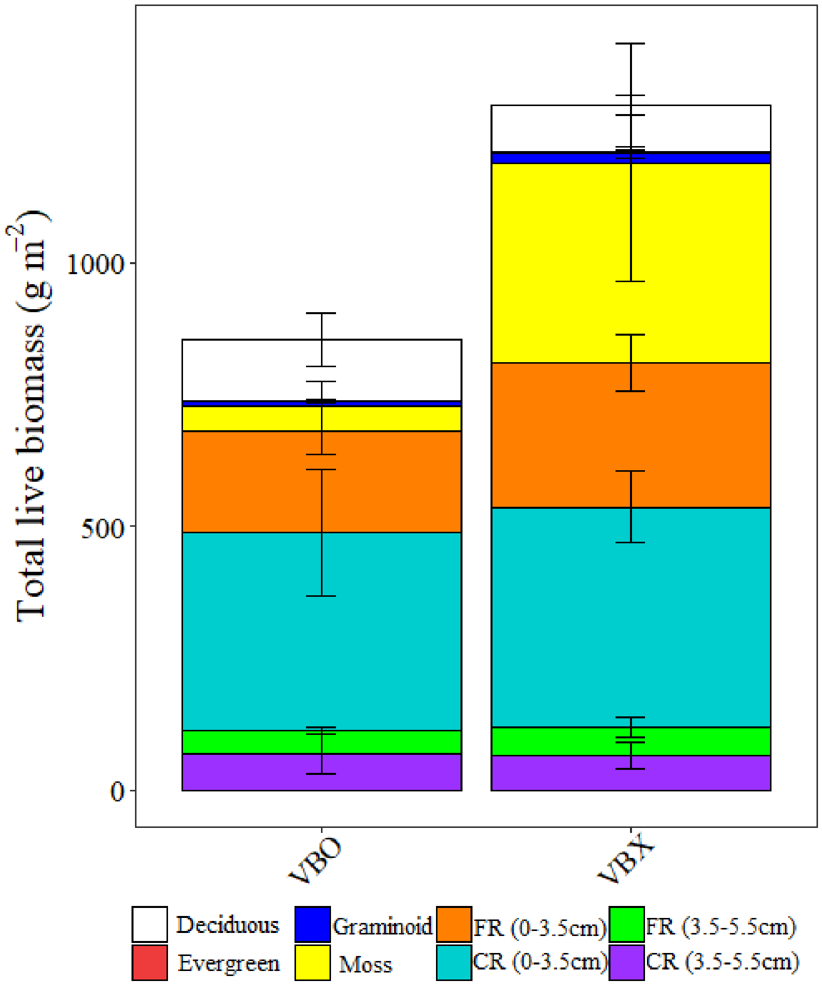

Fig. S1. Biomass of plants by functional forms (deciduous shrubs, evergreen shrubs, graminoids, mosses) and of coarse (CR) and fine roots (FR) from 0-3.5 cm and 3.5-5.5 cm soil depths four years after the fire under ambient temperature (VBO) and warmed conditions (VBX). Error bars are standard errors.

Table S2. Recovery of inorganic ^{15}N and ^{15}N-labelled pyrogenic organic matter (PyOM-^{15}N) in various ecosystem compartments four years after the (simulated) fire under ambient temperature (VBO, VRA) and warmed conditions (VBX, VRX).

^{15}N recovery (%)	Depth	Inorganic ^{15}N		PyOM-^{15}N		
		VBO	VBX	VRA	VRX	
Bulk soil	0-3.5 cm	11.7±5.6	21.8±9.2	11.3±2.0	26.0±13.4	
	3.5-5.5 cm	3.6±0.7	4.9±1.6	2.7±1.0	1.1±0.2	
Coarse root	0-3.5 cm	1.2±0.5	2.8±1.3	0.09±0.04§	0.09±0.02§	Form**
	3.5-5.5 cm	0.2±0.2	0.2±0.2	0.003±0.001§	0.006	Form**
Fine root	0-3.5 cm	2.8±2.0	**3.6±0.7***	0.20±0.07§	0.31±0.09§	Form** Warming**
	3.5-5.5 cm	0.2±0.1	**0.5±0.2***	0.02±0.00§	0.03±0.01§	Form** Warming*
Microbial biomass	0-3.5 cm	0.3±0.1	0.3±0.3	0.001±0.001	0.052±0.023	
	3.5-5.5 cm	0.2±0.1	0.6±0.3	0.021±0.007	0.061±0.050§	Form**
Total dissolved N	0-3.5 cm	0.002±0.001	0.004±0.001	0.005±0.002	0.002±0.000	
	3.5-5.5 cm	0.004±0.001	0.004±0.003	0.002±0.001	0.001±0.001	
Aboveground live biomass		4.2±1.0	9.5±2.5	0.04±0.02§	**1.39±1.07***§	Form** Warming**
Total live biomass[#]		8.6±2.7	16.5±2.7	0.35±0.11§	**1.80±1.08***§	Form** Warming**
Total ecosystem recovery		32.9±9.2	52.8±12.6	16.1±2.2§	**50.8±12.1***§	Warming**

Numbers indicate mean ^{15}N recovery (± standard error) of replicate blocks (n = 5). Significant differences between ambient temperature and warmed plots are shown as *$p \leq 0.05$, and significant differences between ^{15}N forms are shown as § $p \leq 0.05$. Significant effects of ^{15}N form and warming are shown as * $p \leq 0.05$, ** $p \leq 0.01$.
[#]^{15}N recovery in total live biomass represents the sum of ^{15}N recoveries in fine and coarse root and aboveground live biomass pools.

Table S3. Results of the Mixed Models of warming and ^{15}N form effects on functional group-specific ^{15}N recovery.

Model: plant ^{15}N recovery ~ ^{15}N form * warm + (1|block)			
Deciduous shrub ^{15}N recovery			
	df	F value	*p* value
^{15}N form	20	99.5	$p < 0.01$
warm	20	1.4	0.25
^{15}N form * warm	20	1.7	0.21
	df	t.ratio	*p* value
VBO vs. VRA	18.8	7.1	$p < 0.01$
VBX vs. VRX	18.8	5.5	$p < 0.01$
VBO vs. VBX	18.8	0.08	0.94
VRA vs. VRX	18.8	-1.6	0.14
Evergreen shrub ^{15}N recovery			
	df	F value	*p* value
^{15}N from	20	0.02	0.90
warm	20	0.02	0.90
^{15}N form * warm	20	3.9	0.06
	df	t.ratio	*p* value
VBO vs. VRA	18.8	7.1	$p < 0.01$
VBX vs. VRX	18.8	5.5	$p < 0.01$
VBO vs. VBX	18.8	0.08	0.94
VRA vs. VRX	18.8	-1.6	0.14
Graminoid ^{15}N recovery			
	df	F value	*p* value
^{15}N from	15	9.4	$p < 0.01$
warm	15	4.7	0.047
^{15}N form * warm	15	3.1	0.10
	df	t.ratio	*p* value
VBO vs. VRA	18.8	0.82	0.42
VBX vs. VRX	18.8	3.1	$p < 0.01$
VBO vs. VBX	18.8	-2.5	0.02
VRA vs. VRX	18.8	-0.25	0.81
Moss ^{15}N recovery			
	df	F value	*p* value
^{15}N form	15	0.80	0.38
warm	15	9.8	$p < 0.01$
^{15}N form * warm	15	0.15	0.70
	df	t.ratio	*p* value
VBO vs. VRA	18.8	0.81	0.43
VBX vs. VRX	18.8	0.32	0.76
VBO vs. VBX	18.8	-1.7	0.10
VRA vs. VRX	18.8	-2.2	0.039

Table S4. Results of the Mixed Models of warming and ^{15}N form effects on shrub-specific ^{15}N recovery.

Model: shrub ^{15}N recovery ~ ^{15}N form * warm + (1|block)			
Betula nana			
	df	F value	*p* value
^{15}N form	20	1.8	0.20
warm	20	0.7	0.40
^{15}N form * warm	20	0.7	0.41
	df	t.ratio	*p* value
VBO vs. VRA	18.8	0.3	0.75
VBX vs. VRX	18.8	1.4	0.18
VBO vs. VBX	18.8	-1.1	0.30
VRA vs. VRX	18.8	0	1
Vaccinium uliginosum			
	df	F value	*p* value
^{15}N from	20	44.6	$p < 0.01$
warm	20	0.8	0.39
^{15}N form * warm	20	0.8	0.39
	df	t.ratio	*p* value
VBO vs. VRA	18.8	4.8	$p < 0.01$
VBX vs. VRX	18.8	3.7	$p < 0.01$
VBO vs. VBX	18.8	-0.002	1
VRA vs. VRX	18.8	-1.1	0.28
Salix glauca			
	df	F value	*p* value
^{15}N from	15	10.8	$p < 0.01$
warm	15	0.8	0.37
^{15}N form * warm	15	0.6	0.43
	df	t.ratio	*p* value
VBO vs. VRA	18.8	2.6	0.02
VBX vs. VRX	18.8	1.6	0.13
VBO vs. VBX	18.8	1.1	0.29
VRA vs. VRX	18.8	-0.08	0.94
Pyrola grandiflora			
	df	F value	*p* value
^{15}N form	20	0.1	0.76
warm	20	0.1	0.76
^{15}N form * warm	20	4.1	0.06
	df	t.ratio	*p* value
VBO vs. VRA	18.8	-1.5	0.16
VBX vs. VRX	18.8	1.1	0.29
VBO vs. VBX	18.8	-1.1	0.29
VRA vs. VRX	18.8	1.5	0.16

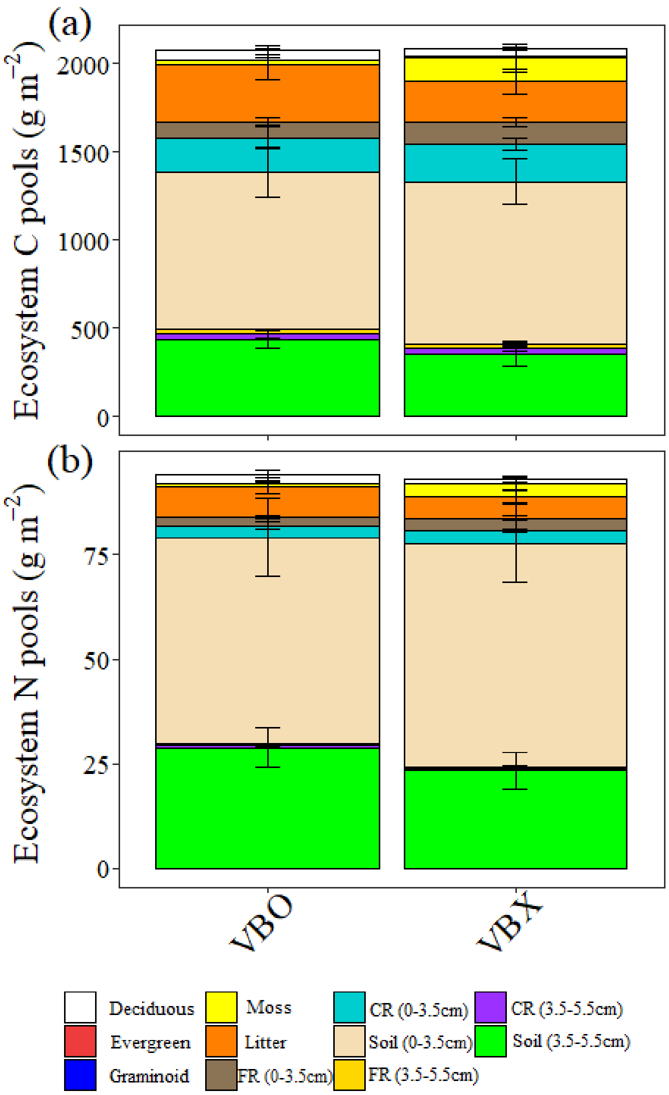

Fig. S2. Carbon and nitrogen contents in plants by functional forms (deciduous shrubs, evergreen shrubs, graminoids, mosses) and in bulk soil, coarse (CR) and fine roots (FR) from 0-3.5 cm and 3.5-5.5 cm soil depths four years after the fire under ambient temperature (VBO) and warmed conditions (VBX). Error bars are standard errors.

Table S5. Recovery of inorganic ^{15}N and ^{15}N-labelled pyrogenic organic matter (PyOM-^{15}N) in the bulk soil and root pools from 0-3.5 cm soil depth during two years after the (simulated) fire under ambient temperature (VBO, VRA) and warmed conditions (VBX, VRX).

		VBO	VBX	VRA	VRX
Soil ^{15}N recovery (%)	2017 (2 days)	28.5±5.6		35.2±4.3	
	2017 (21 days)	22.6±4.7		34.4±16.5	
	2018	30.9±9.7	30.9±6.3	27.1±4.4	30.7±7.2
	2019	27.6±8.8	31.6±15.3	38.2±12.0	33.6±9.7
Root ^{15}N recovery (%)	2017 (21days)	0.3±0.1		0.02±0.005	
	2018	1.3±0.4	3.6±1.8	0.31±0.05	0.47±0.13
	2019	2.2±0.5	3.7±0.8	0.28±0.11	0.69±0.21

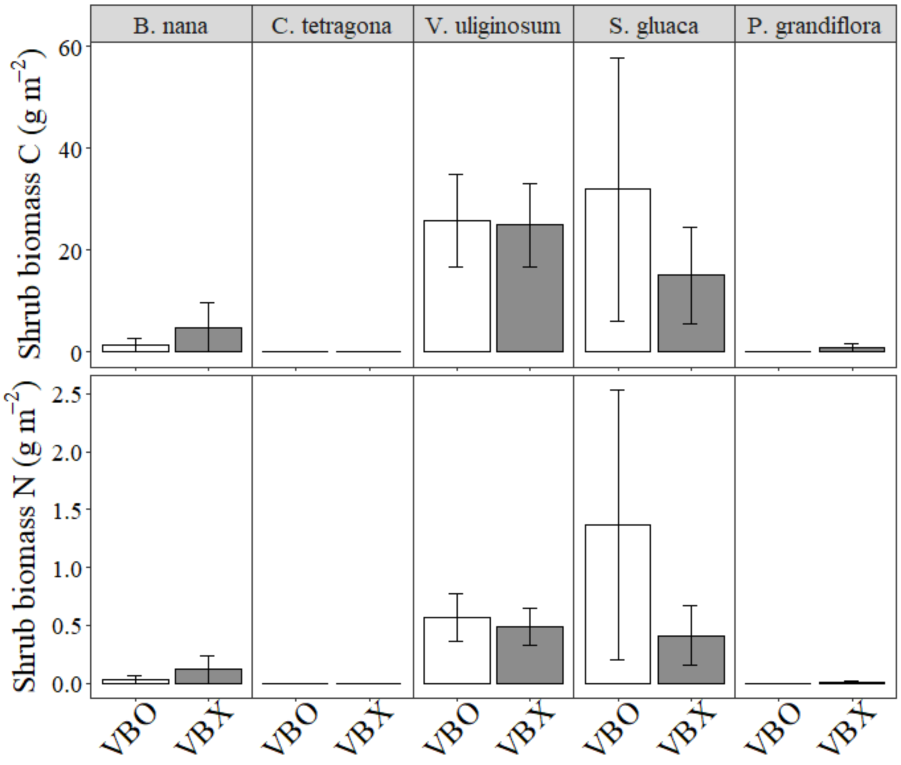

Fig. S3. Species-specific shrub biomass carbon (C) and nitrogen (N) four years after the fire under ambient temperature (VBO) and warmed conditions (VBX). Deciduous (*Betula nana*, *Vaccinium uliginosum* and *Salix glauca*) and evergreen shrubs (*Cassiope tetragona* and *Pyrola grandiflora*) are presented. Error bars are standard errors.

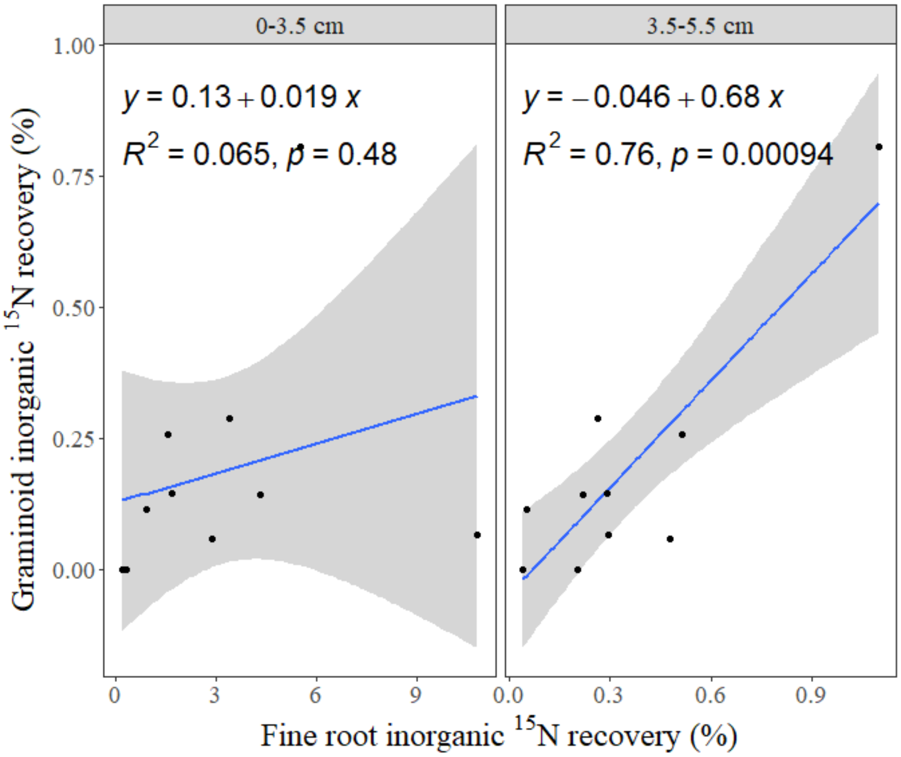

Fig. S4. Relationship between graminoid inorganic ^{15}N recovery and fine root inorganic ^{15}N recovery at 0-3.5 cm and 3.5-5.5 cm soil depths.

Table S6. Enrichment of inorganic ^{15}N and ^{15}N-labelled pyrogenic organic matter (PyOM-^{15}N) in the leaves of the four dominant shrub species during three years after the (simulated) fire under ambient temperature (VBO, VRA) and warmed conditions (VBX, VRX).

		VBO	VBX	VRA	VRX
Betula nana (µg excess ^{15}N g N^{-1})	2018	378±194	86±12	11	0
	2019	36±10	98±14	9	12
	2020	99±41	151±63	28±8	10±1
Cassiope tetragona (µg excess ^{15}N g N^{-1})	2018	0	0	0	0
	2019	0.2	63±51	0	11
	2020	101	103±22	6±1	6±6
Vaccinium uliginosum (µg excess ^{15}N g N^{-1})	2018	505±190	588±242	10±4	10±1
	2019	390±85	289±76	20±3	12±4
	2020	600±338	499±304	20±8	12±5
Salix glauca (µg excess ^{15}N g N^{-1})	2018	691±64	522±170	14±2	23
	2019	360±71	115±96	19±2	24±14
	2020	216±81	190±74	16±4	36±15

No error bars are shown, n < 3 due to no or less plots observed for the species.
